# National perinatal survey demonstrates a decreasing seroprevalence of *Toxoplasma gondii* infection among pregnant women in France, 1995 to 2016: impact for screening policy

**DOI:** 10.2807/1560-7917.ES.2021.26.5.1900710

**Published:** 2021-02-04

**Authors:** Eve Robinson, Henriette de Valk, Isabelle Villena, Yann Le Strat, Mathieu Tourdjman

**Affiliations:** 1 French National Public Health Agency (Santé publique France), Saint-Maurice, France; 2 European Programme for Intervention Epidemiology Training (EPIET), European Centre for Disease Prevention and Control (ECDC), Stockholm, Sweden; 3 National Reference Centre for Toxoplasmosis, Maison Blanche Hospital, University Reims Champagne-Ardenne, France

**Keywords:** toxoplasmosis, Toxoplasma gondii, seroepidemiologic studies, pregnant women, prenatal diagnosis

## Abstract

**Background:**

Toxoplasmosis during pregnancy can result in congenital anomalies or fetal death. Universal antenatal screening is recommended in France, a strategy in place since the 1970s.

**Aim:**

We determined the seroprevalence of toxoplasmosis among pregnant women participating in the 2016 national perinatal survey (ENP), compared results with previous ENPs, and investigated factors associated with *Toxoplasma gondii* infection.

**Methods:**

Using the 2016 ENP data, which contain sociodemographic and clinical information from all women giving birth during a one week period, we calculated adjusted prevalence ratios (aPR) by sociodemographic factors. Using available data from prior ENPs (1995, 2003 and 2010), we calculated age-standardised seroprevalences and aPRs for French women.

**Results:**

In 2016, seroprevalence was 31.3% overall. Among French women, associations with increasing age (aPR: 1.54; 95% CI: 1.39–1.70), residence in Paris (aPR: 1.19; 95% CI: 1.08–1.31) or south-western regions (aPR: 1.19; 95% CI: 1.08–1.31), and higher professional status (aPR: 1.12; 95%CI 1.04–1.21) were observed. An association with increasing age was also evident among women from North Africa and sub-Saharan Africa. Age-standardised seroprevalence decreased from 55.0% in 1995 to 33.7% in 2016. Among French women, significant associations with age, Paris and south-west regions persisted across all ENPs.

**Conclusion:**

Higher prevalences in older women may reflect a higher past risk of exposure while persistent geographical differences may reflect dietary or environmental differences. *Toxoplasma* seroprevalence among pregnant women continues to fall and will impact screening effectiveness. This warrants a comprehensive review to determine the appropriate future of prevention in France.

## Introduction

Toxoplasmosis is an infection caused by the protozoan parasite *Toxoplasma gondii*, which is present worldwide. Felids are the definitive hosts and warm blooded animals are intermediate hosts. The most common route of transmission to humans is by ingestion of tissue cysts in the undercooked meat of other intermediate hosts [1]. Felids excrete oocysts in their faeces, which are infectious within days — after sporulation in the external environment. Humans and other intermediate hosts can also be infected through consumption of fruits, vegetables, or water contaminated with oocysts [2]. Inadvertent ingestion of oocysts after contact with contaminated soil is a less frequent source of infection, while direct contact with cat faeces, for example during pet care, is a potential, but probably rare source of infection.

In the majority of people, acquired infection (i.e. infection after birth) is asymptomatic or causes a mild illness with influenza-like symptoms. However, it can lead to severe disease, particularly among people who are immunocompromised or when infected with a particularly virulent strain [3]. Primary infection in pregnancy can result in vertical transmission (i.e. transmission to the fetus) and congenital toxoplasmosis. Toxoplasmosis has been ranked as one of the highest contributors to the burden of food-borne disease [4-7], with congenital toxoplasmosis accounting for a high proportion of its burden [8].

Vertical transmission is estimated to occur in 25% of maternal antenatal infections overall, although the risk increases with gestational age [9]. Conversely, the risk of severe congenital toxoplasmosis is highest with infection early in pregnancy. Congenital disease is characterised by ocular, visceral or intracranial lesions, which can lead to fetal death or severe sequelae for the child such as visual problems, seizures and learning disabilities. The majority of babies born with congenital infection are asymptomatic. However, ocular disease, typically chorioretinitis, may not be manifest until months after birth, and sometimes not until adolescence or later. Although prenatal treatment in the case of maternal infection is standard practice, there is no strong evidence of its efficacy in reducing congenital infection or the severity of congenital disease, and there is a lack of international consensus on the best mitigation strategy [10,11].

In France, a nationally representative surveillance programme for congenital infection, *ToxoSurv*, has been in place since 2007 [12-14]. A network of laboratories report congenital infections diagnosed antenatally or postnatally (up to 1 year of age) to the French National Reference Centre for Toxoplasmosis. Between 2007 and 2018 the rate of congenital infections ranged between 0.2 and 0.3 per 1,000 livebirths (i.e. 151‒240 confirmed cases of congenital toxoplasmosis annually among approximately 800,000 live births).

France has traditionally been considered a high prevalence country, with seroprevalences over 80% reported in the 1960s [15]. Due to this, a congenital toxoplasmosis prevention programme was introduced in 1978. It involves universal first trimester screening with subsequent prevention advice and monthly screening for seronegative women, and antenatal treatment in the event of a seroconversion. While the results of antenatal screening are not systematically available for surveillance purposes, toxoplasmosis among pregnant women has been periodically monitored through national perinatal surveys (Enquêtes nationales périnatales (ENPs)). Between 1995 and 2010 these have shown a decreasing seroprevance from 54% to 37%, and a decreasing incidence of seroconversions from 5.4 to 2.1 per 1,000 pregnancies at risk (i.e. pregnancies where the mother was non-immune at the start of the pregnancy) [16,17].

Our objective was to determine the seroprevalence of toxoplasmosis among pregnant women using the most recent ENP, undertaken in 2016, to identify factors associated with *Toxoplasma* infection, and to compare the results with previous ENPs.

## Methods

### Study population and national perinatal survey

The ENP is a periodic cross-sectional survey of births in France conducted in 1995, 2003, and 2010. The methodology is detailed elsewhere [17-19]. In brief, in the 2016 survey, all births greater than 22 weeks gestation and/or a birthweight over 500 g in all public and private maternity units between 14 and 22 March 2016 were eligible for inclusion. A face-to-face interview post-delivery collected information on a range of sociodemographic and pregnancy-related factors while data collectors extracted specified medical information from clinical records, including toxoplasmosis-related data.

Stillbirths, terminations of pregnancy (TOP), births by minors (<18 years), or where the mother was medically unfit to participate, were excluded from full participation. In such a case, or in the case of refusal to participate, a minimum dataset of non-identifying birth-related indicators was collected from clinical records as authorised by the national data protection agency. Secret births (a regulated situation in France where a woman does not reveal her identity and wishes for the newborn to be made a ward of the state) were excluded.

Previous ENPs followed similar protocols. Prior to 2016, births to minors and stillbirths were not excluded from full participation. Of note, the overseas department of Martinique did not participate in 2010 due to a lack of personnel, and Mayotte did not participate until 2016, after becoming an overseas department in 2011. Specifically in relation to toxoplamosis, the 2016 ENP included only the serological status based on the last toxoplasmosis test during pregnancy (categorised as: absence of antibodies to *T. gondii*, IgG to *T. gondii* present, seroconversion during this pregnancy, or unknown). Previous ENPs recorded the dates of the last negative and the first positive serological tests in the event of a seroconversion.

### Determination of toxoplasmosis serological status

We classified a woman as seropositive if she was documented as having IgG antibodies present or she was documented as having seroconverted during the pregnancy, and seronegative if she was documented as having no antibodies. Of note, analyses of previous ENPs defined a seroconversion only when dates for the last negative and the first positive test were available.

### Data analysis

We included women with a known toxoplasmosis serological status. We calculated the seroprevalence by sociodemographic characteristics and compared differences using chi-squared tests. Univariable and multivariable analysis (UVA and MVA) was undertaken to estimate prevalence ratios (PRs) and evaluate statistically significant factors associated with seropositivity. Due to interactions between age and nationality, we stratified according to self-reported nationality grouped into French women, women from North Africa and women from sub-Saharan Africa (SSA) (categories used in the ENP questionnaire). In the MVA, regions in mainland France were grouped by Zone d’Etudes et d’Aménagement du Territoire (study and regional planning zone; ZEAT), equivalent to the European Union Nomenclature of territorial units for statistics (NUTS) level 1, and overseas departments were combined [20]. We constructed the MVA model using a backward stepwise elimination procedure starting with those variables that had a revealed p value of < 0.01 in the UVA. We used a Poisson model with robust error variance. This model with robust error variance is a recommended alternative to estimating prevalence ratios [21]. The MVA model constructed for French women was then applied to the other nationality groupings. When interpreting results, we considered estimates with a p value of < 0.05 as significant.

Available datasets for the 1995, 2003, and 2010 ENPs included the common variables: serological status, age, gravidity, nationality, level of educational achievement and region of residence. We compared the seroprevalences between ENPs through direct age-standardisation, using females ages 15—44 years from the 2014 census as the reference population. We used MVA stratified by ENP and nationality to compare factors associated with seropositivity over time.

We used STATA version 14.0 (StataCorp, Texas, United States (US)) to analyse the data and QGIS version 2.18 (Open Source Geospatial Foundation, Oregon, US) to generate maps.

### Ethical statement

The 2016 ENP was approved by the French National Council for Statistical Information (number 2016X703SA), the French Data Protection Authority (CNIL; number 915197) and the ethics committee of the French National Institute for Health and Medical Research (IRB00003888 number 14–191).

## Results

### Study population

**Figure 1:**
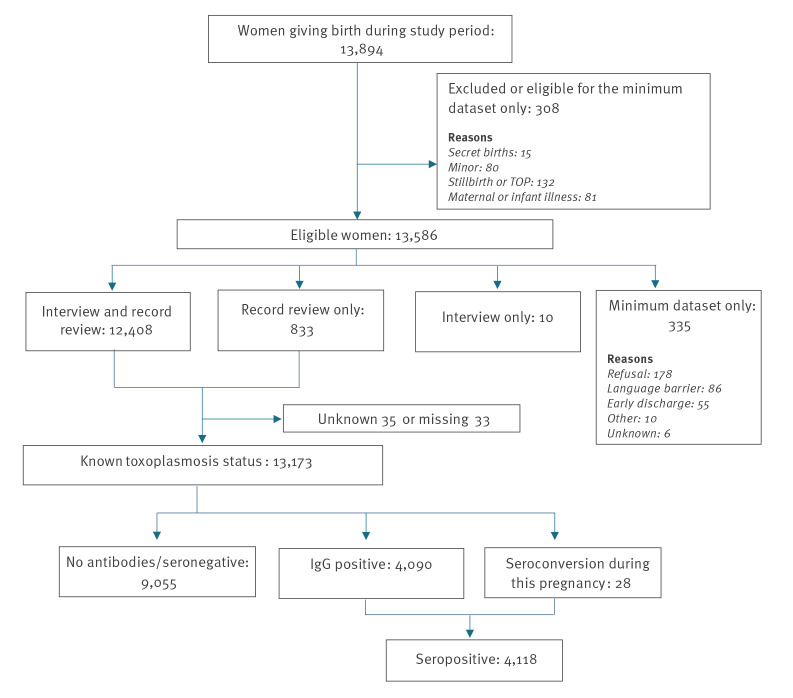
Flowchart of the national perinatal survey population, France, 2016 (n = 13,173)

During the 2016 study week, 13,586 eligible women delivered in participating units (Figure 1). Among these, information on toxoplasmosis serological status was available for 13,173 women. This represents 94.8% of women delivering during the study week and 97.0% of eligible women. Only four of the 517 eligible maternity units, which together had ca 120 births per week during 2016, did not participate.

Among women with a known serological status, median age was 30 years (range: 18‒47 years), 29.4% were primigravida and 85.1% were French (Table 1). Age category was the only demographic variable available to compare women with a known and unknown serological status. There was a significantly higher proportion of women under 20 years than over 20 years among those with an unknown serological status (14.0% vs 1.7%, p < 0.01).

**Table 1:** Demographic characteristics of the national perinatal survey population, toxoplasmosis seroprevalence and crude prevalence ratios by demographic factors, France, 2016 (n = 13,173)

Demographics	Participants		Seroprevalence	Crude prevalence ratio	95% CI	p value
	n	%	%			
Total	13,173	NA	31.3			
**Age (years)**						
< 20	226	1.7	27.4	0.81	0.66–1.01	< 0.001
20–24	1,630	12.4	19.5	0.58	0.52–0.64	
25–29	4,116	31.3	25.0	0.74	0.69–0.79	
30–35	4,417	33.6	33.7	Ref	NA	
35–39	2,248	17.1	42.2	1.25	1.17–1.34	
≥ 40	526	4.0	51.7	1.54	1.40–1.68	
Total (N)	13,163	100.0	NA	NA	NA	
**Gravidity**						
First pregnancy	3,869	29.4	27.5	Ref	NA	< 0.001
≥ 2	9,293	70.6	32.8	1.2	1.13–1.28	
Total (N)	13,162	100.0	NA	NA	NA	
**Educational attainment**						
Primary or less	276	2.3	45.7	1.44	1.24–1.63	< 0.001
Lower second level	2,637	21.5	30.5	0.95	0.89–1.01	
Higher second level	2,684	21.9	28.3	0.88	0.82–0.94	
Third level	6,651	54.3	32.1	Ref	NA	
Total (N)	12,248	100.0	NA	NA	NA	
**Nationality**						
French	10,482	85.1	30.1	Ref	NA	< 0.001
Other European	415	3.4	28.2	0.98	0.80–1.10	
North Africa	583	4.7	38.9	1.3	1.17–1.44	
Sub-Saharan Africa	531	4.3	48.8	1.62	1.48–1.78	
Other	310	2.5	29.0	0.97	0.81–1.15	
Total (N)	12,321	100.0	NA	NA	NA	
**Cohabiting with partner**						
Yes	11,120	90.2	30.8	Ref	NA	< 0.001
No	1,208	9.8	35.7	1.16	1.07–1.26	
Total (N)	12,328	100.0	NA	NA	NA	
**Professional status of the households**						
Without a profession	341	2.8	32.0	1.07	0.91–1.26	< 0.001
Manual	989	8.1	28.8	0.96	0.86–1.07	
Employee	3,499	28.7	28.6	0.95	0.89–1.02	
Intermediate profession	3,734	30.7	29.9	Ref	NA	
Higher profession	2,297	18.9	35.4	1.18	1.10–1.28	
Farmer/ commerce	1,313	10.8	34.8	1.16	1.06–1.27	
Total (N)	12,173	100	NA	NA	NA	
**Monthly income (EUR)**						
< 1,500	2,440	20.2	32.8	1.13	1.07–1.22	< 0.001
1,500–4,000	7,521	62.1	28.7	Ref	NA	
> 4,000	2,142	17.7	36.8	1.28	1.20–1.37	
Total (N)	12,103	100.0	NA	NA	NA	
**Region**						
Bretagne	559	4.8	29.3	0.82	0.71–0.94	< 0.001
Normandie	621	5.3	30.6	0.85	0.75–0.97	
Haut-de-France	1,169	10.0	26.8	0.75	0.67–0.83	
Grand Est	946	8.1	19.1	0.53	0.46–0.61	
Auvergne-Rhône-Alpes	1,497	12.8	25.8	0.72	0.65–0.80	
Bourgogne Franche-Comté	433	3.7	24.7	0.69	0.58–0.82	
Centre Val-de-Loire	458	3.9	31.0	0.87	0.75–1.00	
Pays de la Loire	730	6.3	28.6	0.80	0.71–0.91	
Provence-Alpes-Côte d'Azur	767	6.6	32.1	0.90	0.80–1.00	
Ile-de-France	2,698	23.1	35.8	Ref	NA	
Nouvelle-Aquitaine	891	7.6	33.8	0.94	0.85–1.05	
Occitanie	891	7.6	35.1	0.98	0.89–1.09	
Overseas departments^a^	635	5.4	50.7	1.42	1.29–1.55	
Total (N)	11,660	100.0	NA	NA	NA	

CI: confidence interval; EUR: euros; Ref: reference category used in the analysis.

^a^ Guadeloupe, French Guyana, La Réunion, Martinique and Mayotte combined.

### Toxoplasmosis serological status

**Figure 2:**
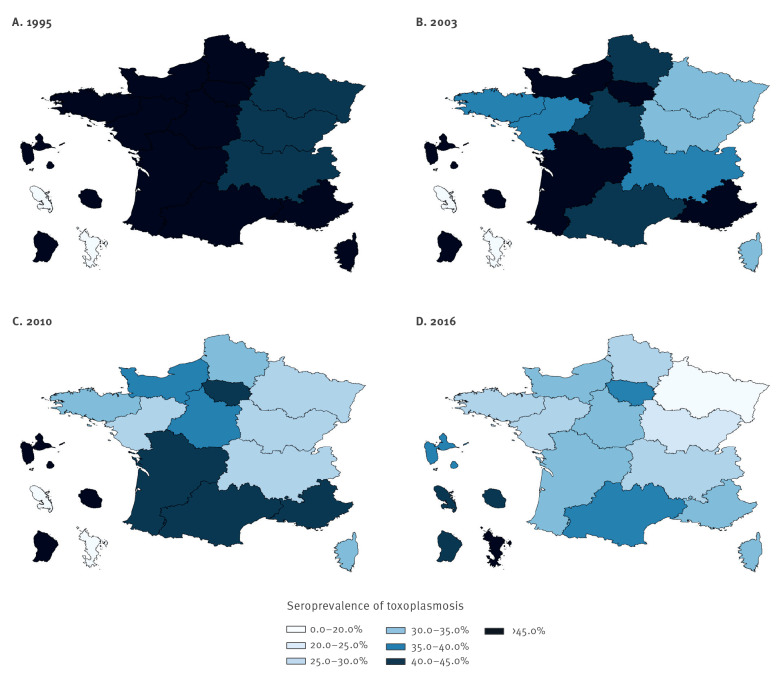
Seroprevalence of toxoplasmosis among pregnant women in France by region of residence as reported by national perinatal surveys performed in (A) 1995, (B) 2003, (C) 2010, (D) 2016

The overall seroprevalence in 2016 was 31.3%. Apart from the youngest age category of under 20 where the seroprevalence was 27.4%, it increased linearly with each age category from 19.5% among women aged 20 to 24 years to 51.7% among women aged over 40 years (p for trend < 0.001). There were significant differences by region with the highest seroprevalences in overseas departments, which had a combined seroprevalence of 50.7% (Guadeloupe 37.3%, French Guyana 41.7%, La Réunion 44.9%, Martinique 43.7%, and Mayotte 76.0%) (Table 1). In mainland France the overall seroprevalence was 30.2%. Here, the highest seroprevalences were in the Paris region (Ile-de-France 35.8%), and south-western regions (Occitanie 35.1%, and Nouvelle-Aquitaine 33.8%), while eastern regions had the lowest seroprevalences (Grand Est 19.1%, Bourgogne-Franche-Comté 24.7% and Auvergne-Rhône-Alpes 25.8%) (Figure 2). The highest seroprevalence of 48.8% was seen among women from SSA compared with 38.9% among women from North Africa and 30.1% among French women (p < 0.001). When restricted to women resident in mainland France the seroprevalence was 40.2% among women from SSA, 39.1% among women from North Africa, and 29.5% among French women. The seroprevalence was also significantly higher among multigravid women (32.8%); women not living with a partner (35.7%); women in either high-income (36.8%) or low-income (32.8%) households; and women from households of a higher professional status (35.4%) or farmer/ commerce professions (34.8%) (Table 1).

Only a combined seroprevalence was available for overseas departments between 1995 and 2010.

Among French women the same associations regarding older age, multigravidity (p < 0.001), the Paris, south and south-western regions and overseas departments (p < 0.001) as described previously, were evident on UVA. There was a statistically significant difference in the seroprevalence by household income, household professional status and educational level (p < 0.001 for all). The seroprevalence was higher in high-income households (crude PR (cPR): 1.34; 95% CI: 1.25–1.44) and among higher professional households (cPR: 1.24; 95% CI: 1.15–1.34). It was lower in those with only second level education (cPR: 0.85; 95% CI: 0.79–0.92) and among manual (cPR: 0.83; 95% CI: 0.7–0.95) and employee (cPR: 0.90; 95% CI: 0.83–0.98) professional households. On MVA, associations with age, region and higher professional status persisted (Table 2).

**Table 2:** Toxoplasmosis seroprevalence by demographic factor and stratified by self-reported nationality, as recorded by national perinatal survey, France, 2016 (n = 13,173)

Demographics	French women				North African women				Sub-Saharan African women			
	Seroprevalence		aPR	95% CI	Seroprevalence		aPR	95% CI	Seroprevalence		aPR	95% CI
	n/N	%			n/N	%			n/N	%		
**Age (years)**												
< 20	31/158	19.6	0.59	**0.41‒0.83**	NA	NA	NA	NA	16/22	72.7	1.19	0.89–1.60
20–24	205/1,249	16.4	0.50	**0.44–0.57**	17/57	29.8	0.72	0.46–1.13	40/83	48.2	0.93	0.69–1.24
25–29	794/3,341	23.8	0.73	**0.67–0.78**	55/180	30.6	0.78	0.59–1.04	68/156	43.6	0.93	0.72–1.20
30–35	1,203/3,606	33.4	Ref	NA	71/179	39.7	Ref	NA	70/160	43.8	Ref	NA
35–39	708/1,732	40.9	1.20	**1.11–1.29**	62/132	47.0	1.16	0.90–1.50	52/88	59.1	**1.43**	**1.12**–**1.84**
≥ 40	210/395	53.2	1.54	**1.39–1.70**	22/35	62.9	**1.50**	**1.10–2.05**	13/22	59.1	1.27	0.87–1.86
**Professional status of household**												
Without a profession	62/238	26.1	1.03	0.82–1.28	10/27	37.0	1.09	0.63–1.89	22/37	59.5	1.21	0.84–1.75
Manual	171/695	24.6	0.98	0.86–1.13	55/135	40.7	1.25	0.90–1.75	29/69	42.0	0.84	0.58–1.21
Employee	788/2,942	26.8	0.99	0.91–1.07	68/158	43.0	1.24	0.91–1.70	78/192	40.6	0.89	0.66–1.20
Intermediate professional	1,012/3,410	29.7	Ref	NA	37/106	34.9	Ref	NA	31/68	45.6	Ref	NA
Higher professional	754/2,052	36.7	1.12	**1.04–1.21**	18/66	27.3	0.89	0.56–1.41	13/33	39.4	0.92	0.56–1.50
Farmer/ commerce	334/1,047	31.9	1.08	0.98–1.19	3277	41.6	1.29	0.89–1.87	58/88	65.9	1.05	0.77–1.43
**ZEAT**												
East	149/798	18.7	0.67	**0.57–0.79**	13/28	46.4	1.07	0.67–1.73	9/14	64.3	**1.76**	**1.05**–**2.94**
Centre-east	327/1,334	24.5	0.84	**0.75–0.95**	24/62	38.7	0.84	0.54–1.31	12/24	50.0	1.43	0.82–2.48
West	401/1,453	27.6	0.97	0.87–1.09	12/24	50.0	1.14	0.68–1.89	13/28	46.4	1.37	0.80–2.33
North	213/766	27.8	1.05	0.92–1.21	3/36	8.3	**0.07**	**0.01–0.51**	2/8	25.0	0.83	0.24–2.90
Paris basin	471/1,730	27.2	Ref	NA	23/56	41.1	Ref	NA	18/51	35.3	Ref	NA
Mediterranean	339/1,007	33.7	1.16	**1.03–1.30**	30/86	34.9	0.79	0.52–1.20	7/14	50.0	1.31	0.66–2.61
South-west	343/968	35.4	1.21	**1.08–1.36**	24/46	52.2	1.14	0.76–1.71	12/24	50.0	1.42	0.81–2.48
Paris	706/1,940	36.4	1.19	**1.08–1.31**	96/238	40.3	0.96	0.69–1.34	88/238	37.0	1.01	0.67–1.50
Overseas departments^a^	191/440	43.4	1.59	**1.39–1.82**	NA	NA	NA	NA	97/128	75.8	**2.11**	**1.43**–**3.12**

aPR: adjusted prevalence ratio ; CI: confidence interval; Ref: reference category used in the analysis; ZEAT: zones d’étude et d’aménagement du territoire (study and regional planning zone, equivalent to Nomenclature of territorial units for statistics level 1 (NUTS 1)).

Values in bold represent a significant difference p < 0.05.

Only variables which were statistically significant on MVA for any of the strata are shown.

^a^ Guadeloupe, French Guyana, La Réunion, Martinique and Mayotte combined.

Among women from North Africa there was also a significant difference by age (p for trend < 0.001) and multigravidity (p = 0.002) on UVA. The seroprevalence differed significantly by educational level (p = 0.02), being highest in those with a lower second level education (cPR: 1.49; 95% CI: 1.17–1.91). The seroprevalence was highest in lower income households (43.2% vs 20.0% in high-income households), although the difference was not of statistical significance (p = 0.06). There were no significant differences in seroprevalence by time since arrival in France (p = 0.7). On MVA, only an association with increasing age persisted (Table 2).

Among women from SSA the seroprevalence again differed significantly by age (p = 0.02), being highest in the youngest and the older age groups. The seroprevalence also differed significantly according to household income (p < 0.001), household professional status and region (p < 0.001), being higher in lower income households (cPR: 1.3; 95% CI: 1.05–1.65), and those resident in overseas departments. The seroprevalence was higher in lower educated women but the difference was not statistically significant (p = 0.07). On MVA, only an association with older age and residence in overseas departments (adjusted PR (aPR): 2.11; 95% CI: 1.43–3.12) persisted (Table 2).

### Seroconversions

Twenty-eight women were documented as seroconverting. Assuming that seroconversions were stable through the year, this corresponds to an incidence of possible seroconversions of 3.1 (95% CI: 1.9–4.2) per 1,000 pregnancies at risk.

### Comparison with previous national perinatal surveys

Study populations of previous ENPs are described in detail elsewhere [17,19]. Age-adjusted seroprevalences were 55.0% in 1995, 44.9% in 2003, 37.7% in 2010 and 33.7% in 2016. In all regions of mainland France absolute decreases in the seroprevalence of between 18% to 28% were seen (Figure 2). In overseas departments the seroprevalence decreased from 61.4% in 1995 to 45.4% in 2010 but then increased to 50.7% in 2016 (only a combined seroprevalence for overseas departments was available before 2016). If Mayotte (which was first included in 2016 and where both French and non-French women had seroprevalences over 75%) is excluded from 2016, the seroprevalence in overseas departments decreases to 42.9% in 2016. Between 1995 and 2016 a decrease in seroprevalence of 25.3% occurred among French women and of 12.5% among women from North Africa. Among women from SSA it increased from 41.7% in 1995 to 48.5% in 2003 and then remained stable. Excluding Mayotte (where 22% of women from SSA were resident) from 2016 resulted in a decrease among women from SSA to 41.2%.

On MVA, stratified by ENP and nationality, the association with increasing age and the Paris and south-west regions among French women persisted across all ENPs (Table 3). Among women from North Africa, a significant association with lower educational level was first evident in 2010, and an association with increasing age was not evident until 2016 (data not shown).

**Table 3:** Toxoplasmosis seroprevalence by demographic factors among French women stratified by the different national perinatal surveys, France, 1995-2016

Demographics	National perinatal survey							
	1995		2003		2010		2016	
	aPR	95% CI	aPR	95% CI	aPR	95% CI	aPR	95% CI
**Age (years)**								
20–24	**0.79**	**0.74–0.83**	**0.68**	**0.63–0.73**	**0.58**	**0.53–0.64**	**0.48**	**0.42–0.55**
25–29	**0.89**	**0.85–0.92**	**0.86**	**0.82–0.90**	**0.75**	**0.71–0.80**	**0.71**	**0.66–0.77**
30–35	Ref	NA	Ref	NA	Ref	NA	Ref	NA
35–39	1.04	0.99–1.10	**1.19**	**1.13–1.25**	**1.20**	**1.13–1.28**	**1.20**	**1.12–1.29**
≥ 40	**1.14**	**1.05–1.24**	**1.25**	**1.13–1.37**	**1.35**	**1.22–1.49**	**1.53**	**1.38–1.69**
**Educational attainment**								
Primary or less	1.02	0.92–1.12	**0.84**	**0.70–0.99**	1.07	0.81–1.40	0.97	0.66–1.41
Lower second level	0.97	0.94–1.01	0.97	0.92–1.01	1.03	0.97–1.09	1.03	0.95–1.11
Higher second level	**0.93**	**0.89–0.98**	**0.95**	**0.90–1.00**	0.95	0.89–1.02	0.95	0.88–1.03
Third level	Ref	NA	Ref	NA	Ref	NA	Ref	NA
**ZEAT**								
East	**0.69**	**0.63–0.75**	**0.68**	**0.61–0.75**	**0.74**	**0.66–0.84**	**0.68**	**0.58–0.80**
West	**0.88**	**0.83–0.94**	**0.87**	**0.80–0.94**	**0.88**	**0.80–0.97**	0.98	0.88–1.10
Centre-east	**0.78**	**0.73–0.84**	**0.82**	**0.75–0.89**	**0.76**	**0.68–0.84**	**0.85**	**0.75–0.96**
North	1.03	0.96–1.11	0.98	0.90–1.07	1.03	0.93–1.15	1.05	0.92–1.20
Paris basin	Ref	NA	Ref	NA	Ref	NA	Ref	NA
Mediterranean	1.00	0.94–1.06	1.06	0.98–1.14	**1.11**	**1.01–1.21**	**1.18**	**1.05–1.32**
South-west	**1.11**	**1.04–1.18**	**1.15**	**1.07–1.24**	**1.20**	**1.10–1.32**	**1.22**	**1.09–1.37**
Paris	**1.18**	**1.13–1.25**	**1.23**	**1.16–1.31**	**1.21**	**1.12–1.31**	**1.23**	**1.11–1.35**
Overseas departments^a^	**1.16**	**1.04–1.29**	**1.28**	**1.16–1.41**	**1.38**	**1.22–1.55**	**1.63**	**1.43–1.86**

aPR: adjusted prevalence ratio; CI: confidence interval; Ref: reference category used in the analysis; ZEAT: zones d’étude et d’aménagement du territoire (study and regional planning zone, equivalent to Nomenclature of territorial units for statistics level 1 (NUTS 1)).

Values in bold represent a significant difference p < 0.05.

^a^ Guadeloupe, French Guyana, La Réunion, Martinique and Mayotte combined.

## Discussion

This is the fourth analysis of toxoplasmosis seroprevalence among pregnant women in France using an ENP. The seroprevalence has showed a continuous decrease from 54% in 1995, to 31% in 2016. This is in keeping with modelled estimates that in 2020 the seroprevalence would be 27% [16]. A decreasing seroprevalence, from 65% in 1997 to 55% in 2013, has also recently been reported in a longitudinal study of non-antenatal clinical tests for toxoplasmosis in the Paris region [22]. The higher seroprevalence found in that study is likely to be due to the inclusion of older ages and being conducted in a high prevalence region.

The global seroprevalence of IgG antibodies to *T*. gondii amongst pregnant women is estimated to range from 11.2% in the WHO Western Pacific region to 45.2% in the Americas [23]. Decreasing seroprevalences have been reported from other high-income countries: from 16% to 10% between the 1988–1994 and 2009–2010 periods among 12–49 year olds in the US [24]; from 41% to 26% between 1995–1996 and 2006–2007 among the general population in the Netherlands [25]; from 47% to 22% between 1979–1980 and 2013 among the general population in Portugal [26]; and from 43% to 32% between 1995–2012 among pregnant women in Austria [27]. Decreases have been largely attributed to reduced exposure to contaminated meat due to better husbandry, changes in food storage and preparation (e.g. freezing meat, as an environment of -12 °C for 3 days kills oocysts), and changes in dietary habits [28].

A decrease in exposure through meat is also likely to be responsible for decreases in France. However, this is difficult to demonstrate as there are no representative longitudinal data on toxoplasmosis in animals or contamination of meat. In addition, the risk posed by meat consumption is influenced by complex patterns in trade and consumption. Overall meat consumption in France has decreased, with a particularly notable decrease in sheep meat consumption, previously thought to be a considerable contributor to human toxoplasmosis in France [29-32]. Meat imports have also increased, with France now being a net importer of sheep meat [30]. Meat imported from lower prevalence countries may pose less risk. However, there have been increases in the consumption of some raw meats. Between 2006–2007 and 2014–2015, raw beef consumption increased from 24% to 30% and raw pork consumption increased from 3% to 6% [33]. Despite the high, and increasing, frequency of raw beef consumption, bovine meat has been considered a less important source of infection due to the low prevalence among bovines. Although, modelling studies in the Netherlands and Italy did find bovine meat to be the most important source of meat-derived toxoplasmosis due to high levels of consumption [34,35].

A lower risk from domestic cats, due to a decreased prevalence among cats (attributed to less outdoor exposure and less eating of wild food), and better hygiene practices around cat litter have also been hypothesised as contributing to decreasing seroprevalences [22]. Data from the pet food industry show that nearly a third of French households own a cat [36,37]. While the number of domestic cats in France has increased by over 3 million to 13.5 million between 2000 and 2016, over 70% are house cats and 80% are fed specialised pet food. In addition, contradictory associations have been found between cat ownership and seropositivity [25]. Therefore, any contribution of cat-related factors to the decreasing seroprevalence is likely to be minimal.

The higher seroprevalence among women of non-French nationality found in all ENPs is consistent with higher seroprevalences among foreign-born populations reported by others [24,38]. This is likely due to increased exposure in the country of origin. However, due to the grouping of nationalities from wide geographic regions, our findings regarding non-French nationalities need to be interpreted with caution. The impact of the inclusion of Mayotte, where the majority of immigration is from other Comoro islands, on the seroprevalence among women from SSA in the 2016 ENP illustrates this. We also found differences by socioeconomic factors between French women and non-French women. Among French women the seroprevalence increased with socioeconomic status, while among women from North Africa and SSA it was highest in the lower socioeconomic groups, although not statistically significantly so. Elsewhere, higher seroprevalences have been found in lower socioeconomic groups [24,25]. Among French women, the association is likely related to dietary habits as those from higher socioeconomic classes are more likely to eat certain undercooked meats [33]. The geographical differences within mainland France have been consistent since 1995, and may be due to dietary habits or to climatic factors which are favourable to persistence of oocysts in the environment [19].

The ongoing decreasing seroprevalence has a number of implications. Firstly, knowledge of the epidemiology is important to guide policy in relation to prevention of congenital toxoplasmosis. The decreasing seroprevalence means more women will be susceptible to infection during pregnancy. However, the infection pressure appears to be lower meaning less risk of exposure. The number of seroconversions during pregnancy decreased from 5.4 per 1,000 at-risk pregnancies in 1995 to 2.1 in 2010 [16]. While we estimated 3.1 possible seroconversions per 1,000 at-risk pregnancies in 2016 this estimate is likely less valid than previous analyses due to changes in data collection and thus cannot be reliably compared. However, modelling, which accurately predicted the seroprevalence trend, had estimated further decreases in the incidence to 1.6 per 1,000 susceptible women by 2020 [16]. While severe congenital disease has certainly decreased since the 1970s it is difficult to demonstrate a corresponding decrease in congenital infections as there are no nationally representative data until 2007 ­– the year the congenital toxoplasmosis surveillance programme, *ToxoSurv*, commenced [12,13]. A network of laboratories report congenital infections diagnosed antenatally or postnatally (up to 1 year of age) to the National Reference Centre for Toxoplasmosis. Between 2007 and 2018 the rate of congenital infections ranged between 0.2 and 0.3 per 1,000 livebirths (i.e. 151‒240 confirmed cases of congenital toxoplasmosis annually among the approximate 800,000 livebirths) [14]. There have been between 19 and 42 cases of congenital infection with either moderate or severe anomalies at birth, or stillbirths or voluntary terminations of pregnancy in the presence of fetal anomalies.

France is one of the few countries in the world offering universal antenatal screening and there is ongoing international debate about its effectiveness [39-41]. In recent years, the United Kingdom reaffirmed its recommendation not to offer antenatal or neonatal screening, stating a lack of understanding on the natural history, test reliability, lack of clear evidence that prenatal or neonatal treatment reduces transmission or severe congenital infection, and concerns about adverse effects of treatments as factors averse to screening [42,43]. Professional societies in North America also recommend against antenatal screening [44,45]. The effectiveness of prevention advice to seronegative women is also of uncertain or limited benefit. A Cochrane review which included two randomised control trials (RCTs), one of which was conducted in France, concluded that there is little evidence that prenatal education is effective in reducing congenital infection [46,47]. Other studies have shown that the incidence is lower among pregnant women compared with non-pregnant women, suggesting differences in risk behaviours potentially due to education [16]. However, the magnitude of the difference in incidence is small. It was estimated that only two of seven congenital infections in Austria would have been prevented by prenatal education [27].

Notwithstanding the clinical efficacy of prenatal treatment in the event of seroconversion or prenatal education, which is beyond our scope to discuss further, the changing epidemiology will impact the effectiveness of the French screening strategy. The decreasing seroprevalence may impact on the predictive value of screening with potential parental anguish or potentially ill informed decisions being made on the future of the pregnancy while awaiting confirmatory testing. The main impact of the decreasing seroprevalence and infection pressures is that, based on the current policy, an increasing number of tests will be undertaken and fewer seroconversions detected. Thus the health benefit achieved by the strategy will lessen, while the costs will increase.

The economic cost and cost-effectiveness of the French policy has not been formally evaluated. It was estimated that in 2008, based on a seroprevalence of 38%, the cost was EUR 43 million per year and that the decreasing seroprevalence was increasing the cost by EUR 1 million per year due to additional monthly testing [48]. One French study, using a seroprevalence of 36.7%, estimated an additional cost of EUR 232,631 per direct toxoplasmosis-related event avoided when comparing current prenatal screening to a potential neonatal screening scenario [49]. When a wider range of adverse events was included the cost per outcome avoided reduced to EUR 14,826. A comparison with a no-screening scenario was not undertaken. An economic evaluation from Austria, which also offers universal screening but with bimonthly follow-up of seronegative women, determined that compared with a no screening scenario, antenatal screening was cost-saving based on a societal perspective and lifetime costs [50]. It is not known how other alternative scenarios would alter the cost-effectiveness of screening. It has previously been shown that changing the testing interval or testing procedure could reduce the cost of screening in France [48]. It is also not known how the cost-effectiveness of the current toxoplasmosis prevention programme compares to other maternal or child health interventions. The ongoing changes warrant a comprehensive evaluation, including an economic evaluation, in order to make an informed decision about the most efficient approach to minimise congenital infections and their sequelae into the future.

Another important result from this study is that, although decreased, the seroprevalence in France is still higher than that reported by some other high-income countries. This suggests that there is room for further control measures to reduce acquired infections, which account for the majority of the burden of disease, in the general population. As mentioned, the effectiveness of health education may be limited. Targeting the sources of infection may be a more effective strategy [28]. However, this requires knowledge on the contribution of different sources to the disease burden, which likely differs by country and which may change over time. As meat is likely to be the predominant source of infection in France, further reducing infection through preharvest or premarket postharvest interventions would likely have the greatest impact. As yet, a vaccine is only available for preventing abortions in sheep and its efficacy in preventing tissue cysts is not known [51]. While rearing practices are known to prevent animal infection, a growing consumer preference for free-range meats may limit reductions in meat transmission [52,53]. Pre-market freezing, particularly of high-risk meats, may also be effective though may not be acceptable to consumers. Any such strategies are currently somewhat limited by the lack of standardised and approved testing protocols in animals and meat products. Any change in the congenital toxoplasmosis screening policy should be considered alongside a renewed focus on wider prevention strategies [54].

### Strengths and limitations

A strength of our study is that it includes data from four nationally representative studies, employing the same methodology, over a 20 year period. A limitation is that the serological status was based on routine testing. As France relies on a large network of private laboratories it is likely that different testing methods with different diagnostic sensitivities were used. Changes in the diagnostic sensitivity of tests over time should also be considered when interpreting temporal trends. Results were extracted from medical records by the ENP data collectors and inter-observer variability in how results were interpreted may exist. Lastly, behavioural risk factors with a plausible causal relationship to toxoplasmosis infection could not be explored as such questions were not included in the ENP questionnaire.

### Conclusion

*Toxoplasma* infection seroprevalence among pregnant women in France has continuously decreased from 80% in the 1960s to 31% in 2016. This changing epidemiology needs to be considered by policymakers, along with other relevant clinical and economic factors, in determining the appropriate future of congenital toxoplasmosis prevention and the screening programme in France.
